# Mortuaries: Their Equipment and Management

**Published:** 1916-03-18

**Authors:** 


### Mortuaries: Their Equipment and Management.

By A HOSPITAL MATRON.

There is no department of the hospital which more needs the care and attention of the matron and sisters than the hospital mortuary. The structure itself cannot be said to fall within the matron's province, she cannot blame herself if it is badly planned, or ugly, and yet not a few of the beautiful little mortuary chapels, whose stained-glass windows by their rich colouring help much to rob the sight of death of its dread, are the result of the matron's efforts to provide a place where mourners can be alone with their dead, amid peaceful and inspiring surroundings. There are, it must be admitted, still many mortuaries which do not fulfil these conditions, and the temptation to leave such a one untended because it cannot be made ideal must not be yielded to: for there is no mortuary in the kingdom that cannot be made less depressing by means of fresh distemper on the walls, and a little table covered with a linen cloth on which is placed a cross and some flowers. A pillow is perhaps one of the most effective of mortuary appointments, for it helps greatly to give a restful appearance to the body—it should not be too soft and should be covered with waterproof material, which is, of course, hidden by the linen pillow-case. The pall of heavy unbleached linen, adorned with a Maltese cross of either blue or red linen, and bordered with the same material, should be turned back by the sister, without whom no mourner should ever visit the mortuary, and should display the head resting on the pillow, the face covered with a hemstitched handkerchief on which a cross is embroidered. The body is, if a man's, clad in a white cotton shirt of cheap material, but neatly finished (such as can be purchased for 2s. 9d.). In the case of a woman a nearly worn-out nightgown is used. A narrow linen sheet is folded under the arms and to it is pinned a neat black-edged card, on which the patient's name and the time of death are inscribed. If the body is that of a child a flower may be placed in the hands.

Money to buy the flowers, furniture, linen, etc., can be obtained by the matron if she makes a practice whenever the mortuary purse needs filling of searching the obituary notices in the local paper and writing to the bereaved persons asking for a gift for the purpose; such a request will rarely remain unanswered. Gifts of linen and nightgowns will often come from the same source. That a matron is repaid for the trouble she has taken cannot be doubted, for the mourners when they have seen how much care has been taken of the body of their loved one are often willing to forgo their custom of having the body taken home to a tiny cottage—a most insanitary and undesirable proceeding; and in any case appreciation is always shown by the mourners; for the poor, who are so extravagant in their funeral arrangements and care so much for the bodies of their loved ones after death, often seem more grateful for what is done in the mortuary than for the efforts that have been made to save the life of which they have been witness in the wards.

*For Appointments see page iv.*

